# Dynamic Interactions of Transcription Factors and Enhancer Reprogramming in Cancer Progression

**DOI:** 10.3389/fonc.2021.753051

**Published:** 2021-09-20

**Authors:** Emily Zboril, Hannah Yoo, Lizhen Chen, Zhijie Liu

**Affiliations:** ^1^Department of Molecular Medicine, Mays Cancer Center, University of Texas Health Science Center at San Antonio, San Antonio, TX, United States; ^2^Department of Cell Systems and Anatomy, Barshop Institute for Longevity and Aging Studies, University of Texas Health Science Center at San Antonio, San Antonio, TX, United States

**Keywords:** enhancers, transcription factors, epigenetics, cancer progression, therapy resistance, metastasis

## Abstract

While improved tumor treatment has significantly reduced the overall mortality rates, invasive progression including recurrence, therapy resistance and metastasis contributes to the majority of deaths caused by cancer. Enhancers are essential distal DNA regulatory elements that control temporal- or spatial-specific gene expression patterns during development and other biological processes. Genome-wide sequencing has revealed frequent alterations of enhancers in cancers and reprogramming of distal enhancers has emerged as one of the important features for tumors. In this review, we will discuss tumor progression-associated enhancer dynamics, its transcription factor (TF) drivers and how enhancer reprogramming modulates gene expression during cancer invasive progression. Additionally, we will explore recent advancements in contemporary technology including single-cell sequencing, spatial transcriptomics and CUT&RUN, which have permitted integrated studies of enhancer reprogramming *in vivo*. Given the essential roles of enhancer dynamics and its drivers in controlling cancer progression and treatment outcome, understanding these changes will be paramount in mitigating invasive events and discovering novel therapeutic targets.

## Introduction

Cancer is the second leading cause of death in the United States. Over 1.8 million new cases and over 600,000 deaths due to cancer were estimated during 2020 (1). Many patients who are declared cancer free following initial treatment may still encounter relapse of disease, which is often more aggressive and frequently results in death. While advancement in treatment has significantly reduced overall mortality rates, progression of disease associated with therapy resistance and metastasis still contributes to the majority of deaths caused by cancer. As cells become malignant, it is imperative they survive and persist, which drives changes in their behavior. Cancer progression is defined by changes in gene expression by which cancer cells are able to survive, metastasize and evade treatment. Cancer progression is marked by the epithelial to mesenchymal phenotypic transition which drives metastasis and treatment resistance (2–4).

Despite the development of new therapeutics, the current most popular option for cancer treatment is chemotherapy. However, prolonged exposure to chemotherapeutic drugs can lead to drug resistance, which remains the biggest challenge for achieving cures in cancer patients. Drug resistance can be intrinsic (due to individual’s genetic differences) or acquired. Acquired drug resistance is more common compared to inherent drug resistance. Drug resistance can occur through different mechanisms, such as mutations, epigenetic changes, altered gene expression, as well as aberrant post transcriptional and translational modifications (5). Most failures of chemotherapy treatment occur during the invasion and metastasis of cancers related to drug resistance.

Metastasis involves a series of steps including the dissemination of cancer cells from primary tumors to the bloodstream, seeding at distant organs and formation of macro-metastasis (6). In the initial phase of metastasis, cancer cells acquire enhanced mobility and invasiveness, and the extracellular matrix undergoes degeneration. After entry into the circulation, tumor cells can disseminate throughout the body and are known as circulating tumor cells (CTCs) prior to colonization. During the colonization phase, disseminated tumor cells (DTCs) often maintain an indolent state, which is characterized by a non-proliferation status, due to unfavorable host microenvironments with immune surveillance as a major defense against metastasis. A combination of intrinsic and extrinsic factors can trigger the exit of the indolent state and activate a proliferation program. These cellular changes can be regulated by specific transcription factors (TFs), although the downstream transcriptional and epigenetic programs governing this change are unclear.

Gene transcription is regulated through the integrated action of many cis-regulatory elements, including promoters and enhancers. Enhancers are localized at greater distances from the transcription start sites (TSSs) and are essential for controlling gene expression (7). Enhancers are typically a few hundred base pairs in length and these small segments of DNA serve as operational platforms to recruit TFs to regulate transcription (8). Multiple TFs often function in an integrated and combinatorial manner on enhancers and the interactions among the TFs are important for enhancer regulation. Genome-wide sequencing has revealed frequent alterations of enhancers in cancers and the reprogramming of enhancers has emerged as one of the important features for tumor (9). Here, we review our current understanding of cancer progression-associated enhancer dynamics and its TF drivers, as well as how we can target enhancers for cancer intervention.

## Enhancer Function and Regulation

### Features of Transcriptional Enhancers

Enhancers are essential distal DNA regulatory elements that control temporal- or spatial-specific gene expression patterns during development and other biological processes (7, 10). Because enhancers can function from many base pairs away from their target, discovering enhancer elements remains challenging (11). For many decades, enhancers have been defined predominately based on functional assays, such as reporter-based assays in transiently transfected cells or transgenic reporters. These methods often use compact elements that can function autonomously. However, *in vivo* enhancer function is much more complex. It’s feasible to define a minimal enhancer, the shortest pieces of DNA that can drive and expression pattern that mimics the endogenous expression pattern. But to achieve a robust stereotypic gene expression that can resist variation in natural conditions requires additional elements besides the minimal enhancer (8). More recent genomic approaches to identify enhancer elements are often based on the following: genome-wide analysis of chromatin accessibility, enrichment of epigenetic markers such as H3K4me1 and H3K27ac, and cooperative binding of TFs, coactivators and RNA Polymerase II (RNA Pol II); assessment of transcriptional potential by global-run on sequencing (GRO-seq) or cap analysis of gene expression (CAGE); or observation of chromatin looping and remodeling to identify physical connections of promoter-regulatory element pairs (12, 13).

Active enhancers are enriched for several chromatin modifications, including H3K27ac and H3K4me (14). H3K27ac, which is catalyzed by p300 and CBP, is commonly used as a marker for active enhancers. A family of MLL complexes can methylate H3K4, resulting in H3K4me1 enriched on enhancers and H3K4me3 enriched on promoters. And the ratio of H3K4me1 to H3K4me3 has been used to identify enhancers over promoters (15–18). However, H3K4 methylation often correlates with transcription activity, with H3K4me3 associated with highly activated promoters and H3K4me1 associated with lowly active promoters (19–22). After enhancers are disengaged or decommissioned H3K4me1 is lost or reduced at enhancers (23). However, in some cases, enhancers may retain H3K4me1 after inactivation (24–26). These H3K4me1+H3K27ac- regions are not necessarily “poised” or “primed” for activation but are sites that are decommissioned. Therefore, combining multiple approaches are generally required to annotate active enhancers.

Enhancers are bound by Pol II and are actively transcribed, generating noncoding enhancer RNAs (eRNA) (27–29). eRNAs are short transcripts ranged in size from 50 to 2000 nucleotides and are often transcribed bi-directionally (28). eRNA transcription has also been suggested to regulate enhancer marker deposition at *de novo* enhancers (30). Currently, a complete definition of eRNA has not been agreed upon, as most eRNA are bi-directionally transcribed, non-polyadenylated transcripts which remain un-spliced, a more stable eRNA has also been discovered which is spliced, polyadenylated and is transcribed unidirectionally (27, 31). Interestingly, it has been observed that knockdown or overexpression of eRNA has a direct effect on target gene expression, suggesting a critical, functional role of eRNA (32). It has been suggested that eRNA may act as a negative regulator of transcription complex assembly (33). While the direct function of eRNA has yet to be agreed upon, mounting evidence suggests that eRNA production is directly correlated to enhancer activity (34). In this way, eRNA may provide the most effective predictive marker for enhancer activity.

### Transcription Factors and Enhancer Activation

Active, functional enhancers require a number of proteins to facilitate enhanced transcription of their target genes. These proteins include specific transcription factors, general cofactors, and chromatin remodeling factors. To activate gene expression, the “opening” of specific enhancer binding sites by pioneer factors is the initial step. This is followed by the binding of required proteins, like TFs and coregulators. Additional protein partners are likely recruited and exchanged during different phases of enhancer priming and activation, making this process reliant on hundreds of proteins (35, 36). Generally, transcriptional initiation can be broken into three phases. The first regulatory layer involves the binding of TFs to the regulatory DNA of a target gene. The second involves recruitment of the chromatin-remodeling complex and a histone modifying complex. Finally, in the third phase, the Mediator complex is recruited and links the enhancer complex of TF to the promoter region of the target gene and recruits RNA Pol II to form the pre-initiation complex (37, 38).

The human genome encodes about 2,600 DNA-binding TFs, with about 200 TFs being expressed in each cell type (39, 40). How different TFs cooperate to regulate enhancer networks has long been an open question. TFs typically consist of one or more DNA binding domains (DBDs) and one or more activation domains (ADs). The DBDs are often sequence specific and bind directly to small, 6-12bp regions of enhancer DNA. This low sequence specificity suggests that the simple affinity of individual TFs for DNA cannot be the only mechanism to control enhancer occupancy and to regulate temporal- or spatial-specific gene expression. Indeed, enhancer regions often contain clusters of different TF binding sites, allowing combinatorial occupancy of different TFs. When these TFs are expressed in overlapping cells, the combinatorial binding can achieve discrete and precise transcriptional regulation (8, 41–43). For example, SMAD3 requires different protein partners to bind enhancers in different cell types and under different conditions. It co-occupies enhancers that target genes required for maintenance of cellular identity with OCT4 in embryonic stem (ES) cells. Additionally, SMAD3 is recruited by MYOD1 in myotubes. Interestingly, induced expression of MYOD1 in mouse ES cells resulted in SMAD3 being redirected to occupy new sites of MYOD1 binding in the genome (44). This demonstrates the importance of combinatorial protein-protein interactions in enhancer function, by illustrating the ability of one transcription factor to produce different regulatory effects depending on its binding partners. TFs can also bind to diverse sets of enhancers at different developmental stages or conditions. The relative affinity or number of binding sites for the TFs may contribute to this context-dependent occupancy, as they can affect TF occupancy if the TF concentration varies overtime (40, 45). Alternatively, TF occupancies can depend on chromatin accessibility, partitioning of TFs to specific cellular compartments, as well as cooperation with other DNA-binding proteins (46).

A large amount of genome-wide binding data show that TFs typically co-bind to “hotspot” regions or cluster to short-range genomic regions (36, 47–51). Within the past decades, studies have demonstrated the new concept of super-enhancers (SEs) in various cell types. SEs are described as clusters of enhancers spanning >8-10 kb, occupied by DNA-binding TFs at their cognate binding motifs (52–54). These clustered super-enhancers regulate key transcription units in stem cells and exhibit high levels of coactivators. Oncogene drivers regulated by super-enhancers are also associated with cancers. SEs are typically identified using the Rank Ordering of Super-enhancer (ROSE) algorithm by analyzing active enhancer markers including H3K27ac and mediator complex subunit 1 (MED1) (53). Besides direct binding to DNA, TFs can be recruited in *trans* to either activate or repress specific target genes (55–57). We have previously identified a new category of ERα TF ‘co-activators’, termed MegaTrans TFs, which are recruited by ERα through protein-protein interactions (*trans*-binding) to active ERE enhancers as ‘co-activators’ (58, 59). Megatrans complex can also recruit specific enzymatic machinery to enhancers and is a signature of the most potent functional enhancers.

The assembly of TFs and co-activators on enhancers has been recently proposed to be the physical process of liquid-liquid phase separation (LLPS) (60–63). LLPS is characterized by the separation of a homogenous solution into two phases of high and low concentrations (64–66). In contrast to the structured DBD, ADs of TFs are generally intrinsically disordered in the amino acid sequences. The transcriptional control at enhancers has the features of phase separation that are driven by these intrinsically disordered regions (IDRs). For instance, IDRs of transcriptional cofactors can form liquid separated condensation at active super enhancers in embryonic stem cells (61, 63). IDRs of TFs, cofactors and RNA polymerase II have all been linked to gene regulation (67–70). Phase separation provides a mechanism by which TFs recruit diverse proteins to the chromatin to drive specific gene expression.

## Enhancer Dynamics in Cancer Progression

### Oncogenic Enhancer Activation

Because dysregulation of transcriptional programs is at the core of cancer development, naturally enhancers play an indispensable role in the initiation and progression of many cancer types (71). Studies have identified over 700 genes in the human genome which are linked to cancer (72), but many genetic variants lie outside the coding portion of the genome and fall in enhancer regions. Genetic variants that target enhancers and affect cancer development include single-nucleotide polymorphisms (SNPs), small insertions or deletions (INDELs), large deletions, inversions, and translocation of existing enhancers (73). In most cases, germline variants only have weak effects on gene expression and cancer development, although they often alter the affinity of TFs to their binding sites. Therefore, cancer development usually requires additional somatic mutations like large genomic rearrangements that cause stronger effects on gene expression (74).

SNPs within the existing enhancers can disturb TF-chromatin interactions, inactivating enhancers. This then leads to transcriptional down-regulation of target tumor suppressor genes and promotes tumorigenesis (75, 76). Alternatively, SNPs might result in the gain of extra TF binding sites and thus induce downstream oncogenic gene expression (76). In some cases, SNPs and INDELs can also generate *de novo* binding sites for TFs, resulting in the formation of oncogenic enhancers that misregulate oncogenes (77–79). For example, heterozygous somatic mutations that introduce binding motifs for the TF named MYB are acquired in a subset of T-cell acute lymphoblastic leukemia (T-ALL) cases. This creates a super-enhancer upstream to the TAL1 oncogene. MYB binds to this *de novo* enhancer and recruits enhancer complex components that contain CBP, RUNX1, GATA3 and TAL1 to promote a leukemogenic transcription program (80).

“Enhancer hijacking”, an event of repositioning an enhancer in a new genomic context, can result from long-range chromosomal structural alterations, including large deletions, inversions, and translocations. Enhancer hijacking can activate oncogenic transcription and promote tumorigenesis (81–83). One example is that the reallocation of a GATA2 enhancer element to the ectopic EVI1 site caused by translocations and inversions leads to concomitant EVI1 and GATA2 deregulation in leukemia (84). Genetic variants disrupting enhancer-promoter looping can also contribute to cancer development. CTCF works together with the cohesion complex to regulate genome topology and drive enhancer-promoter looping. The CTCF and cohesion-binding motifs are frequently mutated in cancer cells, leading to defects in enhancer-promoter looping and aberrant gene expression (85).

Alterations of signaling pathways are commonly associated with cancers. Signal-dependent TFs often bind to enhancers to orchestrate enhancer activity and down-stream gene transcriptional program in response to specific signals that control cancer cells growth (73). For instance, NOTCH signaling activates NOTCH-bound distal enhancers to promote MYC oncogene expression to promote cell proliferation in B-cell lymphomas (86). Oncogenic signal pathways can also cause a genome-wide reorganization of enhancer landscape to promote malignant transformation. Chronic Ras-Erk signaling activates RTK and causes dynamic changes in H3K27ac levels at enhancers, including the ones near GATA4 and PRKCB genes. These changes result in aberrant gene expression and promote tumorigenesis (87). Oncogenic signaling can also modulate enhancer function by recruiting transcriptional machinery. A previous study reported that, in response to the deregulation of the Hippo pathway, YAP/TAZ binds to a specific set of enhancers and recruits the mediator complex and CDK9 elongating kinase to modulate transcriptional elongation of growth-promoting genes (88).

### Function of Enhancer Reprogramming in Cancer Progression

Cancer progression is a process by which cancer cells adjust themselves to achieve resistance to targeted therapies and lead to invasion into host tissues resulting in local and metastatic dissemination. Unlike the deep understanding of the genetic and epigenetic mechanisms that initiate tumors, the mechanisms that drive tumor progression, therapy resistance and metastasis are unclear. Although resistance and metastasis are often studied separately, they share substantially overlapping mechanisms. Among them, alteration of epigenetic pathways is an emerging mechanism of cancer progression. Recent studies have discovered that enhancer reprogramming promotes the adaption of cancer cells to intrinsic and extrinsic changes encountered during tumor progression (73).

ERα-bound enhancers are key elements that regulate gene expression during breast cancer growth and progression. Estrogen (E_2_) and its nuclear receptor ERα are critical for the development of luminal subtype breast cancer (89). More than 70% of cases of invasive breast cancer express ERα (ER+) and are treated with endocrine therapies based on menopausal status (90). While patients with ER+ breast cancer receive endocrine therapies for 5 years, >30% eventually develop therapeutic resistance and disease recurrence, a persistent clinical problem (91, 92). ERα influences genes related to cell growth and endocrine response, primarily through interaction with distal enhancers (58, 59). Thus, many commonly prescribed breast cancer treatments target ER, such as tamoxifen and aromatase inhibitors (93). In ER+ breast cancers, endocrine resistance and prognosis have been linked to alterations in the ERα cistrome (94). By characterizing multiple therapy-resistant breast cancer models, previous studies have shown that high FOXA1 activity can reprogram ERα-dependent transcriptome to promote endocrine-resistant cell growth and invasiveness (95). FOXA1 is a pioneer TF that binds to condensed chromatin to facilitate subsequent binding of ERα and other TFs (96). More recently, it has been demonstrated that FOXA1 upregulation in ER positive breast cancer cells drives global enhancer reprogramming to activate prometastatic transcriptional programs. FOXA1 overexpression also promotes the formation of super enhancers that are associated with endocrine resistance (97).

To further understand the mechanisms governing alterations of ERα cistrome and their roles in breast cancer progression, we characterized context-specific ERα enhancers and their associated transcriptional and phenotypic outcomes in endocrine-sensitive and resistant breast cancers (98). We found that endocrine resistance is associated with elevated phenotypic plasticity, with downregulation of luminal/epithelial differentiation markers and upregulation of basal/mesenchymal markers. We observed similar gene expression profiles in clinical breast tumor samples. Using ATAC-seq and ChIP-seq, we detected genome-wide enhancer gain and loss associated with the hormone-resistance transition and identified context-specific enhancers (*GAIN* and *LOSS* enhancers) that are associated with either endocrine-sensitive or resistant cells. This enhancer reprogramming corresponded to the gene expression changes detected by RNA-seq and GRO-seq. Motif analyses identified GATA3 and AP1 motifs as the most enriched ones associated with *LOSS* and *GAIN* enhancers respectively. Remarkably, GATA3 and JUN are among the BioID identified context-specific ERα cofactors. We further demonstrated the cooperative roles of GATA3 and JUN in controlling enhancer reprogramming and cancer therapy resistance (98).

Similar roles of enhancer reprogramming in promoting cancer progression have also been reported in pancreatic ductal adenocarcinoma (PDA). Using an organoid culture model of PDA that recapitulates the main stages of PDA tumor progression, the Vakoc group characterized how the enhancer landscape evolves during PDA progression and identified alterations in enhancer activity associated with metastasis (99). They defined a subset of enhancers that are associated with metastasis using H3K27ac ChIP-seq with organoids derived from normal ducts, primary tumors and metastatic lesions. Their epigenetic profiling revealed prominent gains and losses of enhancer activity associated with the metastatic transition. They also found that FOXA1 overexpression in PDA cell lines promotes *GAIN* enhancer activation and the acquisition of metastatic phenotypes. However, FOXA1 overexpression in organoids is not sufficient to drive enhancer reprogramming. It turns out that FOXA1 requires cooperation with GATA5 to promote GAIN enhancer activation in the organoid culture model (99). Another study from the same group has demonstrated that TF TP63-driven enhancer reprogramming promotes aggressive PDA tumor phenotypes such as enhanced cell motility and invasion (100).

**Figure 1:**
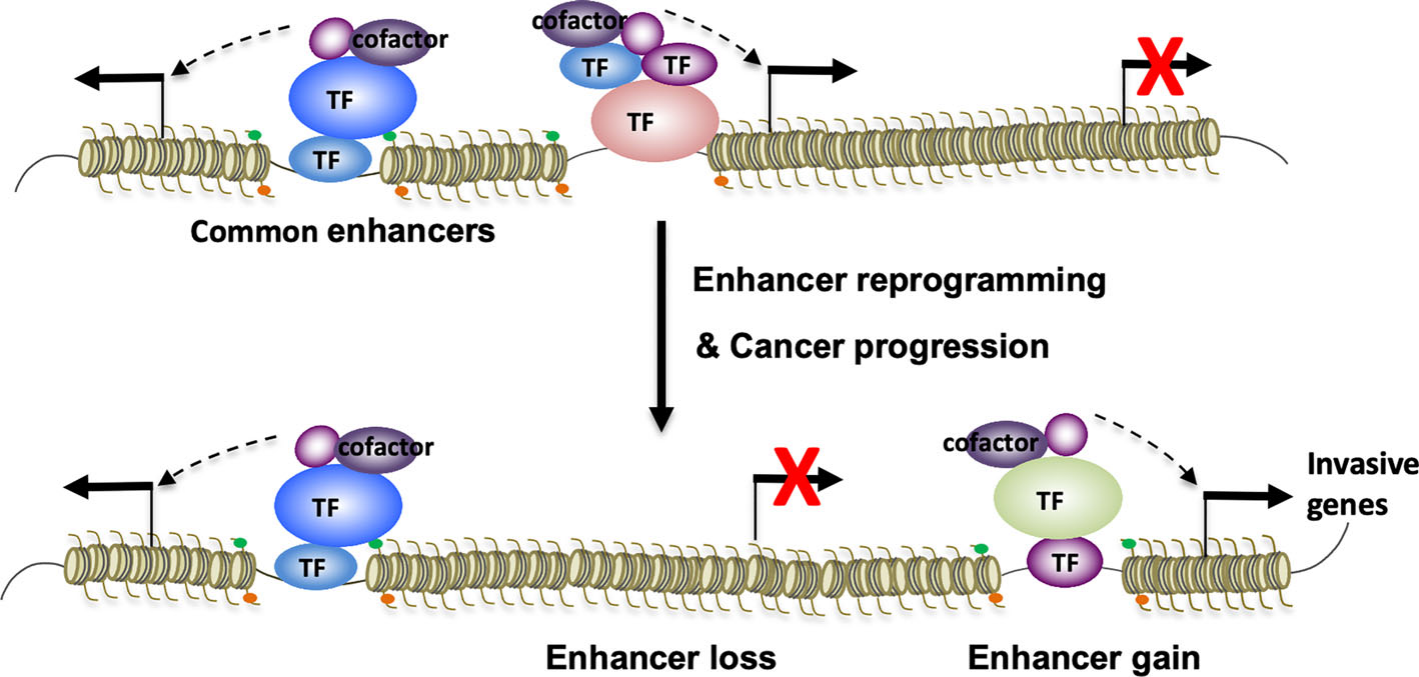
Enhancer reprogramming mediated by context-specific TF-TF interactions promotes lineage plasticity and cancer progression. In response to various signals, interactions of oncogenic TFs on enhancers might undergo context-specific changes. These changes can architecturally reprogram TF-bound enhancers through enhancer loss and gain, resulting in transcriptional transitions that promote lineage plasticity and cancer therapy resistance and metastatic progression.

In an independent study of small cell lung cancer (SCLC) metastasis, TF NFIB overexpression drives metastasis-associated chromatin opening (101). As elevated chromatin accessibility is often associated with enhancer activation, it’s very likely that NFIB can induce enhancer reprogramming to promote SCLC metastasis. Altogether, these studies above lend support to the notion that differential TF assembly on enhancers can lead to global enhancer reprogramming that drives transcriptional transitions and cancer progression (**Figure 1**). Transcription factors that are known to regulate enhancer function and cancer progression are summarized in **Table 1**.

**Table 1:** Summary of known TFs that are involved in enhancer regulation and cancer progression.

Transcription Factor	Role in Cancer Progression
**FOXA1**	Pioneer factor capable of reprogramming ERα-dependent transcriptome to promote endocrine-resistance and invasiveness in breast cancer (97). Overexpression in pancreatic ductal adenocarcinoma (PDA) cells promotes metastatic phenotypes (99).
**GATA3**	Cooperates with JUN to control enhancer reprogramming and therapy resistance in breast cancer (98).
**JUN**	Cooperates with GATA3 to control enhancer reprogramming and therapy resistance in breast cancer (98).
**GATA5**	Cooperates with FOXA1 to drive metastatic reprogramming in PDA (99).
**NFIB**	Drives metastasis associated transcriptional programming in small cell lung cancer (SCLC) (101).
**MYB**	Aberrant binding can recruit enhancer complex components to promote an oncogenic transcriptional program in T-lineage acute lymphoblastic leukemia (T-ALL) (80).
**GATA2**	Deregulation demonstrated in leukemia (84).
**MYC**	Overexpression has been demonstrated to promote proliferation in B-cell lymphomas, this can be induced by NOTCH-signaling (86).
**YAP/TAZ**	Binds enhancers to recruit modulate growth-promoting genes in human cholangiocarcinoma (88).
**TP63**	Drives reprogramming to promote enhanced cell motility and invasion in PDA (100).

## Cancer Treatments Targeting Enhancers

Transcriptional dysregulation is a hallmark of cancer cells. As the growing knowledge of enhancer biology provides us with a more complete understanding of complex gene regulation, we discover more potential targets for therapy. Currently, recommended treatment for most cancer types includes a combination of surgery, radiation, and chemotherapy (102). Unfortunately, these options can result in debilitating acute and chronic side effects and even death (103–105). Because these systemic side effects are often the result of the lack of specificity of therapeutic agents, progressive, targeted treatments must be developed to improve patient care and quality of life during and after treatment. Developing new techniques to fight cancer must include finding targets that are specific to cancer cells, easily influenced or inhibited, and are required by the cancer cells for growth and survival.

Enhancer’s context-specificity makes them an excellent specific target for treatment. Many enhancers are tissue/context dependent and control lineage specific transcriptional programs (106). While specific enhancer programs can vary between cancer types, many share similar qualities: specificity to their cell type, dependence on small molecules that could potentially be manipulated, as well as their necessity for genetic regulation perpetuating malignant programming. Furthermore, a number of contemporary pharmacological treatments targeting enhancers have been shown to be effective. Examples of small molecule inhibitors that have so far been explored target Bromodomain and Extraterminal (BET) proteins as well as cyclin dependent kinases.

Histone modification patterns and their crosstalk work combinatorically with sequence specific binding of transcription-regulating proteins to orchestrate gene expression (107). Epigenetic ‘readers’ are a collection of proteins that have the ability to recognize specific post-translational modifications to histones or DNA (108). Bromodomain-containing 4 (BRD4) is a reader from the BET family of proteins. It contains two bromodomains, which recognize acetylated lysine residues of histones at enhancers and promoters to aid in the recruitment of proteins that facilitate transcription such as the Mediator complex and RNA Pol II (109, 110). Considering the central role enhancers play in the malignant programming of cancer cells, it is unsurprising that targeting BET inhibition (BETi), or BRD4 inhibition specifically, would be an attractive avenue of study. Additionally, many cancer types show dependency on BET mediated transcriptional regulation (111, 112). Although many candidates for BETi compounds have been reviewed, one small molecule inhibitor that has been extensively studied is JQ1. This molecule works by forming a hydrogen bond with the bromodomain of BRD4, rendering it unable to detect acetylated lysine residues on histone proteins, and ablating its ability to recruit transcriptional machinery (113, 114). While there has been some success in the clinical trials of JQ1, there remain concerns regarding its safety and efficacy (115). A deeper understanding of the complex mechanism of BET proteins and their activity regarding enhancer dynamics will be required for success in utilizing BETi technology to influence gene regulation in cancer cells.

Transcription is a tightly regulated process, orchestrated by a plethora of proteins for which there are a number of molecules with non-redundant functions which are required for effective gene expression. For example, cyclin-dependent kinases play a critical role in regulating RNA Pol II activity. Transcription begins by recruitment of RNA Pol II to the promoter-proximal region, a region proximal to the core-promoter that facilitates enhancer-promoter interaction. Here, RNA Pol II begins transcription, but is paused by Negative Elongation Factor (NELF) and BRD-sensitivity Inducing Factor (DSIF) roughly 100 nucleotides downstream (116, 117). Pause-release of RNA Pol II is facilitated by the Positive Transcription Elongation Complex (P-TEFb), which consists of two subunits: cyclin T1 and CDK9. P-TEFb phosphorylates components of NELF, DSIF and RNA Pol II in a process that releases RNA Pol II to begin productive transcription of mRNA (118). Chemical inhibition of this process by attenuating CDK9 function has been shown to not only inhibit transcription of mRNA, but also affect the production of eRNA and enhancer activity (119, 120). Another example of CDK inhibitors is THZ1, a covalent inhibitor of CDK7. While the role of CKD7 is not completely understood, many infer the role of this TFIIH subunit to involve phosphorylating RNA Pol II as well as CDK9 to aid in pause-release and beginning of elongation (121). More recently, additional CDK7 substrates have been identified, such as CDK12 and CDK13, which implicate this molecule as a ‘master regulator’ of transcription (122). Interestingly, THZ1 and other CDK7 inhibitors have been shown to downregulate enhancer associated expression of genes, possibly as a result of decreased enhancer activation (123–125).

While using transcriptional programming as a target for pharmacological modulation presents an exciting, and possibly groundbreaking, avenue for cancer drug development, there remain challenges to designing effective enhancer-targeting treatments. Unfortunately, there has been evidence that BET inhibitor, JQ1, can lead to alternative, compensatory signaling which overcomes inhibition. Studies show JQ1 treatment alters transcriptional programming in castration-resistant prostate cancer such that it becomes BRD4-independent (126). Additionally, the complexity of genetic regulation by enhancers presents a barrier to study in itself. A better understanding of enhancer mediated gene regulation will allow scientists and clinicians to develop safer and more effective cancer treatments.

## Recent Technology Advancements for Studying Enhancer Dynamics *In Vivo*

Technological resolving power is a limiting factor to achieving a more complete understanding of enhancer dynamics. Recent developments have allowed for the study of enhancers *in vivo*, which will garner a more holistic view of dynamic changes that happen during development or disease progression.

Cellular heterogeneity is a fundamental feature of cancer and plays a key role in disease progression and treatment failure (127). Single-cell sequencing has emerged as a valuable tool to address intratumor heterogeneity. Single-cell technology initially focused on single-cell RNA sequencing (scRNA-seq) that can distinguish transcriptional profiles in individual cells and reveal previously unknown cell types or cell states in a complex tissue. Researchers soon realized that many powerful techniques to analyze mRNA and ncRNA production, protein expression, chromatin structure, DNA-protein or protein-protein interactions and more, each of these technologies allows only a one-dimensional view of genetic regulation and expression, and that combination of scRNA-seq with other analyses provides information that is more than the sum of its parts. For instance, simultaneous resolution of mRNA sequences and chromatin accessibility information at the single-cell level can be achieved through Single-Cell Multiome ATAC+Gene Expression assays (128). This allows the detection of multiple data sets from the same cell and studying enhancer dynamics in a more comprehensive way.

*In vivo*, cancer cells locate in close proximity to normal tissue cells, blood vessels, tissue-resident and infiltrating immune cells. The cell-cell interactions within the tumor microenvironment play essential roles in cancer progression. Thus, approaches that can tease apart the complex landscape of tumors will provide insight into cancer biology. Beginning with tissue sections, 10X Genomics Spatial Transcriptomics protocol allows for permeabilization of tissue and barcoding of RNA in specific locations, followed by sequencing. This allows researchers to resolve changes in gene expression in different physical locations. This technology has been utilized to characterize unique interactions between tumor cells and the microenvironment, which would have otherwise been obscured by traditional RNA-seq (129). Other emerging approaches to study cell-cell interactions include, imaging-based mass spectrometry, CyTOF (cytometry by time-of-flight mass spectrometry) and imaging-coupled transcriptional profiling (130, 131).

ChIP-seq has been the predominant method of mapping protein-DNA interactions. One limitation of ChIP-seq is the requirement for large numbers of cells, making it challenging to perform this assay with limited materials, such as tumor tissues. One alternative strategy for ChIP-seq to map protein-DNA interactions genome-wide, termed CUT&RUN, was first reported in 2017 (132). CUT&RUN first attaches unfixed cells to concanavalin A–coated magnetic beads to allow simple handling. Then a specific antibody and a fusion protein composed of protein A and micrococcal nuclease (pA-MN) are introduced to the cells. pA-MN-mediated DNA cleavage will be activated by calcium on the DNA bound by the TF (or other DNA-associated proteins). Cleaved fragments released will diffuse out of the nuclei and be collected to extract DNA directly for sequencing. It has been shown that CUT&RUN can achieve base-pair resolution of TFs with only 10 million sequenced reads, reaching a much higher signal-to-noise ratio than ChIP-seq (133). Overall, CUT&RUN has several advantages compared to ChIP-seq: 1) lower requirement for cell numbers, greatly facilitating enhancer mapping in tissues; 2) simple procedure without chromatin fragmentation or solubilization; 3) low background allowing low sequence depth. Derived from CUT&RUN, CUT&Tag is designed for efficient epigenomic profiling of small samples and single cells (134). CUT&Tag uses a transposome that consists of a hyperactive Tn5 transposase-ProteinA (pA-Tn5) fusion protein loaded with sequencing adapters. After *in situ* tethering by TF-specific antibody and pA-Tn5, Tn5 tagmentation of TF-bound loci generates fragments ready for PCR enrichment and DNA sequencing. The entire process from antibody binding to tagmentation occurs within intact cells. The transposase and chromatin fragments remain bound together, so fragmented DNA is retained within each nucleus, facilitating enhancer analyses at even single-cell level.

## Conclusions and Perspectives

Tumor invasive progression remains the major cause of mortality related to cancer. In addition to genetic alterations, epigenetic changes play a key role in tumor progression by promoting molecular and cellular plasticity. Enhancer function is one of the major drivers in many tumors, as enhancers play a major role in cell identity maintenance and cell adaption to environmental changes, and alterations in enhancer activity can subvert cell fate determination (73, 135). Recent studies have indicated that reprogramming of enhancer function could lead to deregulation of gene expression profile and confer cell growth advantages, promoting cancer progression (97, 99). We also revealed global enhancer reprogramming mediated by differential TF assembly on enhancers drives transcriptional transitions and hormone resistance in breast cancer (98). An essential future goal is to understand the mechanisms that govern enhancer reprogramming in tumor initiation and progression. Enhancer regulation is more than just simple on/off switches and undergoes progressive and regulated changes that are essential for spatial-temporal enhancer function. An emerging mechanism for enhancer regulation is the assembly of transcription machinery as biomolecular condensates on active enhancers. Future studies are required to test whether phase separation of differential TFs on enhancers drives enhancer reprogramming. TFs represent a compelling and biologically validated class of protein targets for pharmacologic intervention in disease, yet their potential remains largely untapped due to the challenges in targeting protein-protein and protein-DNA interactions. Recent successes in targeting protein-protein interactions, including those involving TFs (136), suggest the feasibility of targeting TFs to prevent enhancer reprogramming, thus slowing down or stopping cancer progression.

## Author Contributions

LC and ZL conceived the idea. EZ and HY wrote the draft manuscript. LC and ZL wrote the final manuscript. All authors contributed to the article and approved the submitted version.

## Funding

ZL is a CPRIT Scholar in Cancer Research. This work was supported by funds from CPRIT RR160017 to ZL, V Foundation V2016-017 to ZL, V Foundation DVP2019-018 to ZL, Voelcker Fund Young Investigator Award to ZL, UT Rising STARs Award to ZL, Susan G. Komen CCR Award CCR17483391 to ZL, NCI U54 CA217297/PRJ001 to ZL, NIGMS R01GM137009 to ZL, and Voelcker Fund Young Investigator Award to LC.

## Conflict of Interest

The authors declare that the research was conducted in the absence of any commercial or financial relationships that could be construed as a potential conflict of interest.

## Publisher’s Note

All claims expressed in this article are solely those of the authors and do not necessarily represent those of their affiliated organizations, or those of the publisher, the editors and the reviewers. Any product that may be evaluated in this article, or claim that may be made by its manufacturer, is not guaranteed or endorsed by the publisher.
